# Association of Social Jetlag With Sleep Quality and Autonomic Cardiac Control During Sleep in Young Healthy Men

**DOI:** 10.3389/fnins.2019.00950

**Published:** 2019-09-06

**Authors:** Ágnes Réka Sűdy, Krisztina Ella, Róbert Bódizs, Krisztina Káldi

**Affiliations:** ^1^Department of Physiology, Semmelweis University, Budapest, Hungary; ^2^Institute of Behavioural Sciences, Semmelweis University, Budapest, Hungary; ^3^National Institute of Clinical Neurosciences, Budapest, Hungary; ^4^Department of Laboratory Medicine, Semmelweis University, Budapest, Hungary

**Keywords:** autonomic nervous system, heart rate variability, sleep quality, circadian misalignment, social jetlag, cardiovascular risk factor

## Abstract

Social jetlag (SJL), the difference in sleep timing between work and free days is a consequence of the discrepancy between the individual’s circadian rhythm and the social clock. SJL is considered a chronic stress factor and has been linked to various health problems. In this field study, we examined for the first time the association between SJL and cardiac regulation during sleep. 33 healthy young men aged 20–26 years participated in the study. The median SJL was used as a cut-off value to assign the participants into two groups with either lower or higher SJL. As a marker of autonomic control we analyzed heart rate variability (HRV) and addressed intra-individual differences between workdays and free days. In subjects with higher SJL, pNN50, an indicator of vagal activity was lower in the first 3 h of sleep on workday as compared to free day (day × sleep block × group, *p* = 0.015), indicating a more adaptable regulation on free days, when subjects slept according to their own preference. However, in subjects with lower SJL, no HRV differences were found between the two nights. SJL showed correlation with the free day-workday differences of both pNN50 and another vagal index, RMSSD in the first 2 h of sleep (*p* = 0.023 and 0.047, respectively). In subjects with higher SJL, a different HF power on workdays and free days (*p* = 0.031) also indicated that a shift in sleep timing is accompanied by an altered parasympathetic activity in the first few hours of sleep. Furthermore, subjective sleep quality on workdays was negatively associated with SJL (*p* = 0.02), and subjects with higher SJL reported worse sleep quality on workday than on free day (*p* = 0.027). Taken together, our data call attention on the potential effect of SJL on sleep quality and vagal activity during sleep.

## Introduction

The circadian clock is a fundamental tool enabling organisms to track time internally and thus to adapt their physiology to daily fluctuations in the environment. Circadian time-keeping is organized at the cellular level by the action of molecular oscillators. In mammals, cellular oscillators of peripheral tissues are governed by the central clock located in the suprachiasmatic nucleus of the hypothalamus. A misalignment between the organism’s internal clock and the environmental time is often followed by desynchronization between tissue clocks. Circadian misalignment is considered as a stress factor (Wittmann et al., 2006; Puttonen et al., 2010), and has been linked to the development of various pathological states including cardiovascular diseases (Morris et al., 2017; Strohmaier et al., 2018), metabolic disturbances (Biggi et al., 2008; Vetter et al., 2015), different malignancies (Schernhammer et al., 2006; Papantoniou et al., 2018) and psychological problems (Shields, 2002).

Social jetlag (SJL) is defined as the time difference between the midpoint of sleep on workdays (MSW) and on free days, and it is a consequence of the discrepancy between an individual’s own biological rhythm and the daily timing determined by social constraints (Roenneberg et al., 2003; Wittmann et al., 2006). SJL affects the majority of the adolescent and adult population worldwide. In Europe, more than 30% of the population suffer from an SJL larger than 2 h (Roenneberg et al., 2012). SJL was found to correlate with a higher risk for the development of depression (Levandovski et al., 2011), adverse metabolic changes including obesity and type 2 diabetes (Roenneberg et al., 2012; Rutters et al., 2014; Parsons et al., 2015; Wong et al., 2015; Koopman et al., 2017), and shows association with health-impairing habits such as smoking and excessive caffeine consumption (Wittmann et al., 2006). In addition, our and others’ data indicate that SJL negatively impacts academic performance (Haraszti et al., 2014; Diaz-Morales and Escribano, 2015).

Both epidemiological and laboratory studies indicate that severe forms of circadian misalignment such as shift work or jetlag adversely affect the circulatory system and therefore increase the risk of the development of cardiovascular diseases (Knutsson, 2003; Grimaldi et al., 2016; Morris et al., 2016; Vetter et al., 2016; Hulsegge et al., 2018). SJL is the most frequent type of circadian misalignment, therefore its negative impact on the cardiovascular system would represent a public health concern.

The analysis of heart rate variability (HRV) is a well-established method to assess autonomic cardiac control (Malik, 1996; Laborde et al., 2017). A key advantage of this method is that well-validated programable measuring devices are available for application in field studies without causing any discomfort for the subjects. HRV represents the change in the time interval between successive heartbeats, which is modulated by the sympathetic and parasympathetic nervous system (Malik, 1996). Therefore, HRV is used as a tool to assess autonomic function at the level of the heart. Healthy cardiovascular control is signaled by higher HRV, indicating a stronger parasympathetic activity, whereas low HRV indicates reduced vagal modulation and increased sympathetic activity, which are established risk factors of cardiovascular diseases (Malik, 1996; Thayer et al., 2010). As autonomic control and sleep regulation are interconnected, HRV may be indicative for sleep quality, and efficiency as well. Falling asleep is accompanied by the shift of autonomic balance toward a parasympathetic dominance, measurable in HRV as it increases from wake to slow wave sleep (Elsenbruch et al., 1999; Trinder et al., 2001; Tobaldini et al., 2013; Chouchou and Desseilles, 2014), which is considered to be the most restorative sleep stage (Akerstedt et al., 1997).

In this study, we hypothesized that sleeping out of the endogenous phase on workdays affects cardiovascular regulation and sleep efficiency. To this end we analyzed sleep-related HRV indices and subjective sleep quality in healthy men forming a homogenous sample with respect to age, BMI, and social situation but differing in the extent of SJL, and addressed individual differences between workdays and free days.

## Materials and Methods

### Participants and Protocol

Participants with regular weekly schedule were recruited via advertisements in social media (Facebook groups) and mailing list of university students. Initially 35 students participated in the study, but two of them were excluded either due to experiencing high emotional stress or for lack of appropriate cooperation. Therefore, data of 33 subjects were involved in the final analysis. The sample was homogenous with respect to age (mean ± SD of 23.2 ± 1.5 years), BMI (mean ± SD of 22.8 ± 2.5), and social situation (university students in Budapest). All subjects were healthy and none of them were taking medication. None of the subjects had a history of sleep disorder or cardiovascular problem and neither one of them experienced jetlag in the last month prior to the study. In a short interview, participants were asked about smoking habits, caffeine consumption, physical activity, blood pressure, and cholesterol levels. Answers did not indicate notable cardiovascular risk factors in any participants. Participants were asked not to undergo extreme physical activity, and to avoid drinking alcohol during the study week. Following the measurements, participants were interviewed about any unexpected events (high psychical and physical stress or any health problems). The study protocol was approved by the Semmelweis University Regional and Institutional Committee of Science and Research Ethics (Ethical approval 170/2016). Participants gave written consent prior to entering the study and were paid for their participation. The study took place in winter (2016/2017) and in the first half of spring (2017) and was paused for 2 weeks after changing to daylight saving time. The number of participants with lower and higher SJL was similar in every month. The study for each subject lasted for a week, during which the participants performed their daily activities according to their usual habit, including waking to an alarm clock on workdays but waking on their own on free days. On a workday (Wednesday) and a free day (Saturday) participants wore an ambulatory heart rate recorder (Actiheart, CamNtech Ltd., United Kingdom) during the night and filled out a sleep quality questionnaire upon waking on the next day. On the days of HRV detection, subjects collected urine samples in the evening before going to bed and in the morning directly after getting up.

### Assessment of Sleep Timing

To recruit participants with different SJL and regular weekly schedule, students were asked to fill out the Hungarian version of the Munich Chronotype Questionnaire (MCTQ) (Haraszti et al., 2014). Chronotype was assessed as the midpoint of sleep on free days (MSF) corrected for oversleep on free days (MSFsc), and SJL was calculated by subtracting the midpoint of sleep on workdays (MSW) from MSF, based on Wittmann et al. (2006). During the week of the study each participant kept a sleep diary, based on which the SJL characteristic for the study period was calculated. The mean SJL ± SD was 92.1 ± 52.9 min, which fits well with previously reported SJL values for this age group (Wittmann et al., 2006; Haraszti et al., 2014; Pilz et al., 2018). The median SJL (93 min) was used as a cut-off value to divide the participants into two groups with either lower (SJL ≤ 93 min) or higher SJL (SL > 93 min). Using this cut-off value resulted in a 10-minutes gap between the two groups. There was no significant difference between the two groups regarding age (23.3 ± 1.9 and 23.1 ± 0.9 years mean ± SD for the lower and the higher SJL group, respectively) and BMI (22.1 ± 2.7 and 23.6 ± 2.1 years mean ± SD for the lower and the higher SJL group, respectively). Sleep onset and waking time on the days of HRV detection were estimated by the Actiheart software. These time points were controlled by comparing them with data from the sleep diary. The estimated sleep onset and waking times were used to calculate exact SJL for the days the measurements were performed. This SJL value was used to investigate correlation between SJL and HRV parameters.

### Urinary Melatonin

Urine samples collected by the subjects in the evening and morning were stored at −20°C before analysis. Urinary 6-sulfatoxymelatonin, a stable melatonin metabolite, was measured by competitive ELISA according to the instructions of the manufacturer (IBL International). The creatinine concentration of the samples was determined by enzymatic assay (Diagnosticum Zrt., Budapest) to obtain a normalized 6-sulfatoxymelatonin/creatinine ratio (nmol/mmol).

### HRV Analysis

The Actiheart monitor was used for the recording of heart rate. Prior to the measurements, participants came into the laboratory where a signal test was performed along with the programing of the device to start recording in the evening. On the next day participants brought the device back for data collection and recharge, before taking it again for the weekend. The Actiheart monitor collects inter-beat-interval (IBI) data, which can be further processed and used for the calculation of HRV parameters. Studies comparing Actiheart and standard ECG recordings did not find any significant difference in IBI detection (Brage et al., 2005; Barreira et al., 2009; Kristiansen et al., 2011). All HRV analyses were performed according to the standards of measurements (Malik, 1996) and the recent recommendations by Laborde et al. (2017). First, reliability of raw data was checked by visual inspection. To ensure a consistent artifact detection and correction, the ARTiiFACT software was used (Kaufmann et al., 2011). According to the international guidelines the data were decomposed into 5 min long segments for analysis of HRV parameters in both the time and frequency domains (Malik, 1996). Segments with more than 10% artifact ratio were excluded. Excluded segments and loss of electrode contact resulted in slightly varying sample sizes in the different analyses. Parameters calculated for the 5 min long segments were averaged for either 1.5- or 2-h blocks, depending on the analysis performed, as indicated in the text and legends.

In the time domain the standard deviation of the normal to normal interval (SDNN), the root mean square of successive differences (RMSSD), and the percentage of successive normal to normal intervals differing by more than 50 ms (pNN50) were calculated using the ARTiiFACT software. For the analysis of HRV in the frequency domain the DADiSP 6.7 software (DSP Development Corp., United States) was used. IBI data were first resampled to 4 Hz using cubic spline interpolation, detrended and tapered with a Hanning window. Power spectral density (PSD) was then calculated with mixed-radix Fast Fourier Transformation, yielding 600 frequency bins in the range of 0–2 Hz. From the average PSD of the successive 5 min segments very low frequency (VLF, 0.003–0.04 Hz), low frequency (LF, 0.04–0.15 Hz), and high frequency (HF, 0.15–0.4 Hz) absolute powers were calculated by numerical integration of the respective frequency ranges. Normalized units of LF (LFnu = LF/LF + HF) were also calculated. Bin-wise and band-wise PSD values were log normalized (natural base logarithm) before the statistical analyses. In our subjects respiratory rate, determined on the basis of the peak in the high frequency region of the FFT plot, was between 0.2 and 0.3 Hz (12–18/min). At this respiratory rate (resting state) HF corresponds to vagal activity, and correction of the HRV parameters for respiration is not required (Larsen et al., 2010; Bertsch et al., 2012; Laborde et al., 2017).

### Assessment of Subjective Sleep Quality

For the assessment of subjective sleep quality, we used the Groningen Sleep Quality Scale (GSQS) (Meijman et al., 1988). The Hungarian version of the questionnaire translated from English was validated by Simor et al. (2009). The participants filled out the GSQS questionnaire directly upon waking following the nights with HRV measurements. The questionnaire contains 15 short, true or false questions about the previous night’s sleep. The first question is not evaluated, therefore the GSQS is scored between 0 and 14, a higher score indicating lower quality of sleep.

### Statistics

Normality of data (sleep times, HRV measures, questionnaire scores, and 6-sulfatoxymelatonin levels) was assessed by the Kolmogorov-Smirnov test. 6-sulfatoxymelatonin and bin-wise spectral HRV data showed non-normal distribution. For the analysis of the former the non-parametric Mann-Whitney *U* test was used. Bin-wise spectral HRV data was log normalized to achieve normality. If normal distribution was present, we used parametric statistical procedures as follows. Workday-free day differences were assessed by paired sample *t*-tests. Group differences were tested by two sample *t*-tests. For the analysis of HRV data in consecutive time blocks, repeated measures analysis of variance (ANOVA) was performed. Fisher’s LSD test was used as *post hoc* test. In addition, the association of SJL with sleep quality and HRV was examined by Pearson’s correlation. Log-normalized bin-wise spectral data was expressed as a difference (free day – workday) over the frequency axis (0–0.4 Hz). One-sample *t*-tests were run in order to test if the differences were significantly deviating from the null hypothesis (difference = 0). Inflation of Type-I error in this case was handled by the procedure of descriptive data analysis (Abt, 1987). Statistical significance threshold was set at *p* < 0.05. If not otherwise indicated, data are presented as mean ± standard error of the mean (SEM). Statistical analyses were performed using Statistica software version 13.2 (StatSoft, Tulsa, OK, United States).

## Results

### Basic Rhythm and Sleep Characteristics of the Participants

To control that the participants’ actual sleep schedule during the study week was similar to their usual sleep timing, data from the sleep diary recorded during the study week were compared with data from the MCTQ filled in prior to the study. Reliable correlation for both chronotype (indicated by MSFsc) and SJL were obtained (Supplementary Table S1). Participants were divided into two groups based on the median SJL of the whole sample. Table 1 shows sleep data (timing and duration) for both the workday and free day when subjective sleep quality and HRV were assessed. On workdays the two groups displayed no differences in sleep timing and duration, whereas on free days the group with higher SJL had a significantly later bedtime, sleep onset, and waking time than the other group. Both groups had longer sleep duration on free day compared to workday. As a factor of homeostatic sleep regulation we calculated the time spent awake on the days before the HRV measurements. However, neither a group nor a group x day effect was obtained (Supplementary Table S2).

**TABLE 1:** Sleep characteristics of the two groups of participants on the days of the measurements.

					**Comparisons**	
					
	**Workday**		**Free day**		**Workday**	**Free day**
			
**Time (hh:mm)**	**Lower SJL**	**Higher SJL**	**Lower SJL**	**Higher SJL**	***p***	***p***
Bedtime	23:45 (00:52)	23:51 (00:49)	23:56 (00:48)	01:27 (00:52)	0.775	<0.0001
Sleep onset	23:57 (00:50)	00:03 (00:51)	00:09 (00:50)	01:36 (00:51)	0.713	<0.0001
Waking up	07:09 (00:39)	07:02 (00:28)	08:45 (01:09)	10:00 (01:40)	0.540	0.019
Sleep duration	07:13 (00:56)	06:59 (00:43)	08:35 (01:11)	08:25 (01:27)	0.433	0.706

*Comparison of sleep timing and duration between groups with lower and higher SJL on workday and free day. Bedtime was indicated by entries of the sleep diary, whereas sleep onset and waking up were estimated by the Actiheart software and verified by diary data. Mean (SD), two-sample *t*-test, *n* = 17 and 16 for the group with lower and higher SJL, respectively.*

As a marker of circadian rhythm, urinary levels of 6-sulfatoxymelatonin, the stable metabolite of melatonin, were determined in evening, and morning urinary samples. The marked difference in the levels of 6-sulfatoxymelatonin between the morning and evening samples (mean individual morning/evening ratios ± SEM, 6.75 ± 0.75) confirmed the rhythmic physiology of our participants. However, neither the evening nor the morning excretion of 6-sulfatoxymelatonin was associated with SJL (Supplementary Table S3).

### Time-Domain Analysis of HRV

**FIGURE 1:**
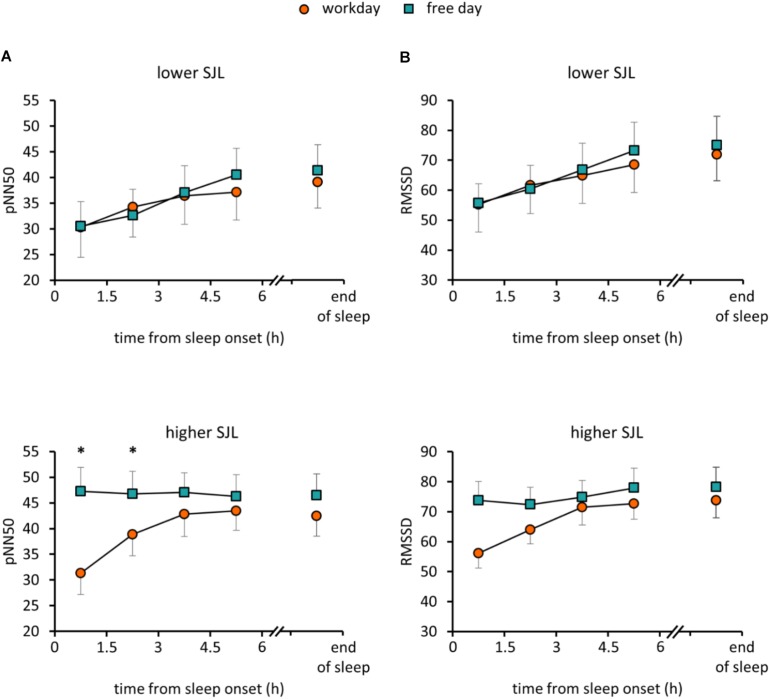
HRV parameters in the course of sleep on workday and free day. PNN50 **(A)** and RMSSD **(B)** values were averaged for 1.5-h sleep blocks on workdays and free days for participants with lower (upper panels) and higher SJL (lower panels). The first four consecutive sleep blocks correspond to the first 6 h of sleep. The last sleep block, presented after an axis break, corresponds to the last 1.5 hour of sleep on the workday and the corresponding sleep segment (same time form sleep onset) on the free day. Analyses for the first 6 h of sleep: Repeated measures ANOVA with three levels (day, sleep block, group), *post hoc* Fisher test, ^∗^*p* < 0.05; analyses for the last sleep block (after the axis break): paired *t*-test; *n* = 15 for the group with lower SJL and *n* = 11 for the group with higher SJL.

To assess how sleep timing affects autonomic cardiac control during sleep, individual HRV parameters were compared between workdays and free days. Average heart rate (HR) for the whole sleep period did not differ between the two days in either group of participants. However, in the group with higher SJL, SDNN, a basic measure of HRV, was significantly higher on the free day than on the workday (Table 2). To analyze HRV in the course of sleep, we calculated mean HRV parameters for consecutive 1.5-h sleep blocks from the time point of falling asleep. As the minimum sleep length on both days was 6 h, four sleep blocks were analyzed for each night and the data sets of workdays and free days were compared. Figure 1 shows data for RMSSD and pNN50, the time-domain parameters most commonly used for the characterization of vagal control of heart function (Malik, 1996; Laborde et al., 2017). Repeated measures of ANOVA with three factors (group, day, and sleep block) revealed no significant effect of group or day, only sleep block showed significance among the main factors (Supplementary Table S4). Furthermore, a day × sleep block × group interaction was obtained (*p* = 0.015; *F*(3,72) = 3.74) for pNN50. While the HRV values nearly overlapped in the group with lower SJL, in the first two time blocks data of subjects with higher SJL showed intra-individual differences as their values were significantly lower on the workday than on the free day (Figure 1A) (higher SJL, workday, first block: 31.3 ± 4.1; second block: 38.9 ± 4.2; free day, first block: 47.3 ± 4.7, second block: 46.8 ± 4.4). *Post hoc* test did not reveal difference between the two groups in any sleep block. RMSSD showed a similar tendency, as values diverged in the first two blocks in the group with higher SJL, however, the interaction did not reach the level of significance [*p* = 0.059; *F*(3,72) = 2.59] (Figure 1B). To exclude that the HRV difference was counteracted by an opposite HRV difference after the sleep period previously analyzed (first 6 h), we performed a further analysis comparing the average HRV in the last 1.5 h of sleep on the workday and in the corresponding sleep segment (same time form sleep onset) on the free day (Figures 1A,B). However, the end-of-sleep HRV data did not display differences between workday and free day.

**TABLE 2:** HR and HRV data for the whole sleep period.

					**Comparisons**	
					
	**Lower SJL**		**Higher SJL**		**Lower SJL**	**Higher SJL**
			
	**Workday**	**Free day**	**Workday**	**Free day**	***p***	***p***
HR (1/min)	59.1 (2.1)	58.1 (1.3)	57.8 (1.8)	57.2 (2.1)	0.520	0.739
SDNN	86.8 (7.3)	95.1 (6.4)	91.5 (6.2)	102.2 (6.9)	0.088	0.018
RMSSD	61.1 (8.3)	64.9 (7.4)	64.1 (4.5)	72.8 (5.4)	0.422	0.092
pNN50	32.8 (5.1)	35.2 (4.3)	37.7 (3.6)	44.2 (3.9)	0.540	0.107

*Mean HR and HRV data were averaged for the time period from sleep onset to waking up. Data for the workday and the free day were compared within both groups of participants. Mean (SEM), paired *t*-test, *n* = 17 and 14 for the group with lower and higher SJL, respectively.*

**FIGURE 2:**
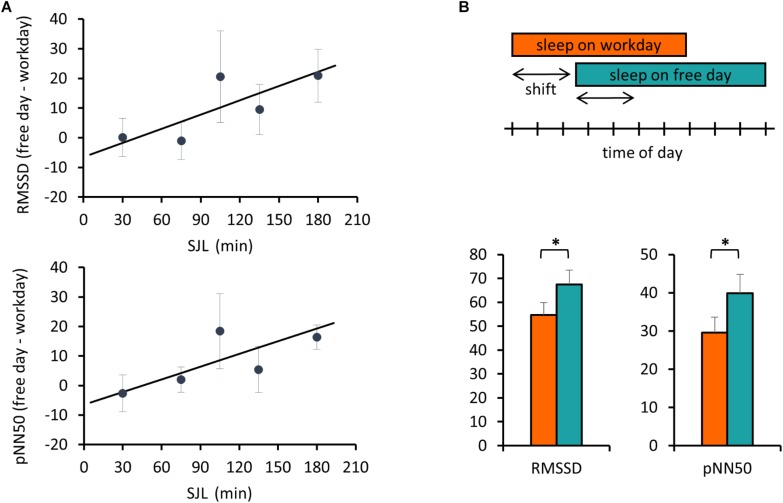
Free day – workday differences in HRV during sleep are associated with SJL. **(A)** Correlation of SJL with the difference between free and workday values of RMSSD and pNN50. RMSSD and pNN50 values were averaged for the first 2 h of sleep. Individual free day – workday differences in RMSSD (upper panel) and pNN50 (lower panel) were calculated and correlated with SJL. For better visualization mean values for SJL intervals of either 30 or – in case of low sample number (first and last two intervals) – 60 min are shown. Trend lines for linear regression are shown (upper panel) Pearson’s *r* = 0.365, *p* = 0.047, *n* = 30 and (lower panel) Pearson’s *r* = 0.4135, *p* = 0.023, *n* = 30. **(B)** Advanced sleep timing on workday is related to a lower HRV. Schematic illustration of the analyzed sleep periods (indicated by the arrows) equal to the shift of sleep onset between work and free day. The boxes represent the time of day from sleep onset until waking (upper panel). RMSSD and pNN50 data were averaged for the shift periods and compared between work and free days (lower panel). Paired *t*-test, ^∗^*p* < 0.05, *n* = 18.

Next, we addressed the relationship between SJL and the HRV difference between the workday and the free day. Analyzing HRV parameters for the first 3 h, corresponding to the first two sleep blocks in Figure 1, a positive correlation between SJL and the free day-workday differences in pNN50 was obtained (Pearson’s *r* = 0.39, *p* = 0.033, *n* = 30). When the analysis was restricted to the first 2 h, both RMSSD and pNN50 differences were positively correlated with SJL (*r* = 0.365, *p* = 0.047, *n* = 30 for RMSSD and *r* = 0.4135, *p* = 0.023, *n* = 30 for pNN50) (Figure 2A).

In the next analysis, we compared the beginning of sleep for a duration equal to the difference of sleep onset between the 2 days, referred as shift of sleep onset (Figure 2B). Data of all participants with more than 25 min shift were involved in this comparison. Both RMSSD and pNN50 parameters were significantly lower on the workday than on the free day (54.7 ± 5.3 and 67.5 ± 6.0, *p* = 0.016 for RMSSD, and 29.6 ± 4.1 and 39.8 ± 5.0, *p* = 0.009 for pNN50).

### Frequency-Domain Analysis of HRV

**FIGURE 3:**
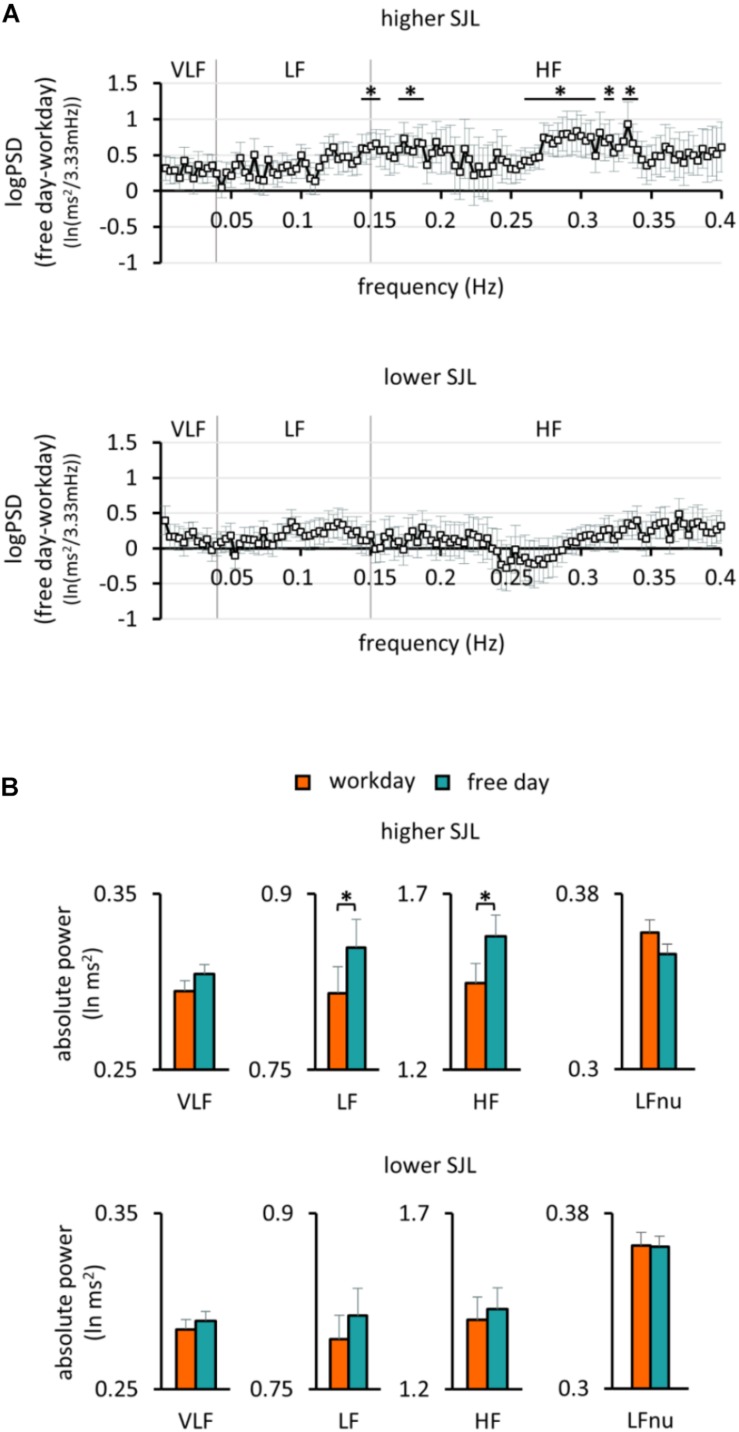
Frequency analysis of HRV in the first 2 h of sleep. **(A)** The bin-wise differences in the power spectral density of HRV between free days and workdays in the group with higher (upper panel) and lower SJL (lower panel). One sample *t*-test, ^∗^*p* < 0.05 indicates above the chance power differences (multiple comparison corrected by the Descriptive Data Analysis protocol), *n* = 13 and 17 for the group with higher and lower SJL, respectively. **(B)** Powers of the standard frequency bands on work and free day in the group with higher (upper panel) and lower SJL (lower panel). Paired *t*-test, ^∗^*p* < 0.05, *n* = 13 and 17 for the group with higher and lower SJL, respectively.

To further characterize the HRV differences in the two groups of participants, frequency domain analyses were also performed. When the PSD of HRV was plotted for the first 2 h of sleep for the work and free day, the curves overlapped in the group with lower SJL, whereas they differed in the group with higher SJL (Supplementary Figure S1). We calculated the individual bin-wise free day – workday differences in the PSD. The difference was significant in several frequency ranges in subjects with higher SJL, but not in those with lower SJL (Figure 3A). The frequency band-based analysis of PSD showed that both LF [0.82 ± 0.02 and 0.85 ± 0.02 (lnms^2^) on workday and free day, respectively, *p* = 0.027] and HF [1.45 ± 0.06 and 1.58 ± 0.06 (lnms^2^) on workday and free day, respectively, *p* = 0.031], but not VLF [0.29 ± 0.01 and 0.30 ± 0.01 (lnms^2^) on workday and free day, *p* = 0.103] bands were involved in the free day elevation of HRV of subjects with higher SJL (Figure 3B). In contrast, no differences between work and free day were found in the group with lower SJL [Figure 3B; VLF, 0.28 ± 0.01 and 0.29 ± 0.01; LF, 0.79 ± 0.02 and 0.81 ± 0.02; HF, 1.40 ± 0.07 and 1.43 ± 0.06 (lnms^2^) on workday and free day, respectively]. To approach sympathetic modulation, normalized units of LF were also calculated. LFnu powers were relatively low as expected during sleep in healthy young people (Brandenberger et al., 2003; Stein and Pu, 2012). Workday and free day LFnu powers did not differ significantly in either group (Figure 3B).

### Examination of the Association Between SJL and Subjective Sleep Quality

**FIGURE 4:**
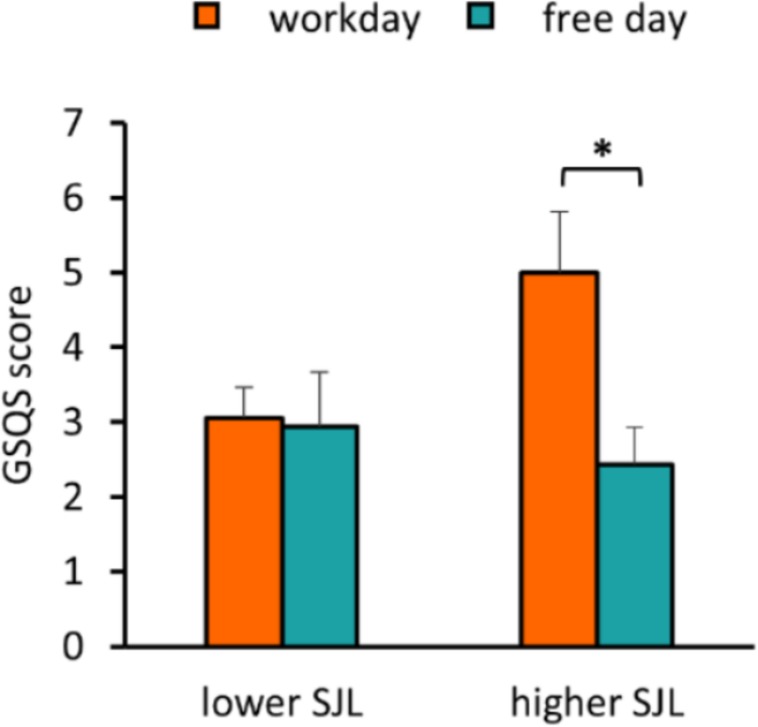
Negative association of SJL with sleep quality. Average workday and free day GSQS scores of the participants in the groups with lower and higher SJL are shown. Paired *t*-test, ^∗^*p* < 0.05, *n* = 17 and 16 for the group with lower and higher SJL, respectively.

To assess sleep quality, we used the GSQS where a higher score indicates worse quality of sleep. We found that SJL was positively associated with the GSQS score on workday, whereas no association was obtained on the free day (Supplementary Figure S2). While participants in the group with lower SJL reported similar sleep quality for both days, participants with higher SJL had a significantly worse sleep quality on the workday than on the free day (*p* = 0.027) (Figure 4), and their workday scores were also significantly higher than those of the other group (two-sample *t*-test, *p* = 0.043).

## Discussion

To our knowledge, this is the first study indicating an interrelationship between SJL and the control of cardiac function. SJL affects most people in our society for shorter or longer periods throughout their life. As a strong and positive correlation between SJL and chronotype was found, and chronotype peaks between ages 20 and 26 years, SJL affects young adults most intensively (Wittmann et al., 2006). Therefore, for this field study we recruited male university students and addressed whether differences in sleep timing between workdays and free days show associations with sleep-related autonomic regulation and subjective sleep quality. Importantly, the measurements were performed in the participants’ home environment and subjects kept their usual sleep schedules both on workdays and on free days. This was confirmed by the correlation of both chronotype (MSFsc) and SJL determined on the basis of the MCTQ (usual sleep timing) and the sleep diary (sleep timing in the study week).

We analyzed HRV, as it is a sensitive marker of the plasticity of the autonomic control of cardiac function. According to literature data, the baseline HRV can show high variations among healthy individuals and the source of these inter-individual differences is largely unclear (Goldberger et al., 2001). Beside the plasticity of autonomic control, other factors such as structural and functional properties of the brain were suggested to influence absolute HRV values (Thayer et al., 2012; Kumral et al., 2019). Therefore, intra-individual HRV changes may be more informative than inter-individual differences in absolute HRV values. Nevertheless, it is interesting to note, that in the group with higher SJL the average HRV was higher in both nights compared to the group with lower SJL. Larger sample and further investigations would be needed to analyze whether a higher HRV during sleep is characteristic for people with higher SJL and/or later chronotype.

When we examined individual changes in HRV between the workday and the free day, we found that in the first 3 h of sleep in the group with higher SJL both RMSSD and pNN50 were lower on workday than on free day. HRV parameters can display circadian variations (Hilton et al., 2000; Vandewalle et al., 2007; Scheer et al., 2009). As we had recordings from only 1/3rd of a day, we cannot assess the daily rhythm of HRV in our subjects. Nevertheless, it might be possible that in the group with higher SJL a decrease in HRV in later sleep segments leads to compensation of the high HRV observed in the first sleep blocks. However, in the end of sleep HRV showed no differences between the 2 days, suggesting that the HRV divergence in the first 3 h of sleep was not counteracted in later sleep segments. A positive correlation between SJL and the free day-workday difference in various HRV parameters suggested that a higher SJL is associated with a larger increase of parasympathetic modulation from the workday to the free day. Results of the frequency-domain analysis were consistent with the time-domain data, as subjects with higher SJL showed different PSD of HRV on free day compared to workday, dominantly in higher frequency regions reflecting the vagal activity (Scholz et al., 1997; Montano et al., 2009; Laborde et al., 2017). Although the sleep duration of most participants was longer on free days than on workdays, this was not dependent on SJL, indicating that there were no differences in sleep deprivation during workdays between the groups of participants. Time spent awake, a main component of homeostatic sleep regulation, was also similar in the two groups. These data suggest that differences in the circadian rather than in the homeostatic sleep regulation are responsible for the workday-free day differences of HRV parameters in people with higher SJL. The apparent deviation of HRV parameters in the first two sleep blocks resulting in a different pattern of HRV in the course of sleep between workday and free day may reflect a difference in sleep structure. In this early period of workday’s night, the biological clock of people with higher SJL (and later chronotype) may not promote sleep. However, the shift of sleep onset from the socially forced earlier time to the preferred later bedtime may cause a deeper sleep with greater vagal tone on free day’s night compared to workday’s night. A recent work showed that timing of sleep onset is associated with changes in the proportion of sleep stages (Akerstedt et al., 2018). In addition, the positive psychical effect of sleep timing without restrictions may also beneficially influence both the autonomic function and the sleep quality of participants with higher SJL.

Altogether, we suggest that SJL and the chronic changes in sleep timing cause a difference in vagal activity between work and free days’ sleep and may affect the plasticity of cardiac regulation in the first few hours of sleep.

HRV parameters were found to reflect the functional properties of the cardiovascular system in both healthy populations and under pathological conditions. In young male subjects a positive correlation was obtained between vagal indices of HRV and endothelial functions (Pinter et al., 2012). Results of a recent meta-analysis based on eight studies with a total number of almost 22 000 subjects showed that low (but still normal) HRV in healthy populations, i.e., without known cardiovascular diseases, is associated with increased risk of a later cardiovascular event (Hillebrand et al., 2013). Moreover, autonomic dysregulation of the heart may contribute to hypertension (Schroeder et al., 2003), coronary artery calcification (Colhoun et al., 2001), arrhythmias, and congestive heart failure (Chen et al., 2014; Florea and Cohn, 2014; Fukuda et al., 2015).

As autonomic control and sleep regulation are interconnected, HRV can serve as an indicator of sleep quality as well. On the other hand, good subjective sleep quality *per se* is considered a marker of both healthy cardiovascular control and emotional wellbeing (Massar et al., 2017; Cespedes Feliciano et al., 2018). Our results based on the GSQS showed that SJL negatively impacts sleep quality on workdays. In a recent study using the Pittsburgh Sleep Quality Index (PSQI), Pilz et al. (2018) found that SJL mediates the effect of chronotype on the differences in sleep quality between workdays and free days. As GSQS and PSQI differ in both the items to be answered and the referred period (while the GSQS refers to the sleep quality of the previous night, the PSQI is an instrument assessing sleep quality over the last month), results of Pilz et al. (2018), and our data are complementary and together strongly indicate that SJL negatively impacts sleep quality on workdays.

Data from this study should be interpreted by considering some limitations. The sample size was relatively low. To exclude a possible effect of cycle-dependent changes in sexual hormones, only men were involved in this study. Therefore, it is possible that the interactions examined are characteristic only for men but not for women. Further studies may examine possible sex differences. In order to have a relative homogenous sample, only young adults were involved in the study. Impact of age on the associations found in our study could be the subject of future investigations.

In summary, we suggest that the chronic changes in sleep-wake patterns due to social constraints are associated with lessening of the restorative capacity of sleep on workdays. This is reflected by the differences of autonomic cardiac control between workdays and free days and the lower sleep quality during workday nights. In addition, our findings provide further support for the recent claim indicating that the cardiovascular system is particularly sensitive to circadian variation (Scheer et al., 2009; Janszky et al., 2012; Grimaldi et al., 2016).

Considering the very high prevalence of SJL in both the adolescent and the adult population, our findings together with literature data about the adverse health effects of SJL stress the requirement to develop social strategies for the reduction of SJL. Even small changes or more flexibility in school and work schedules may lessen the harmful effect of SJL. Due to shifting the phase of the circadian clock, daylight saving time (DST) also aggravates SJL and negatively affects health (Kantermann et al., 2007). Therefore, initiatives to abolish DST are currently under consideration. Individual strategies may also be important tools for the reduction of SJL. For example, increasing morning and decreasing evening light exposure can shift the biological clock to an earlier phase and thus lessen SJL. As meal acts as an effective *Zeitgeber*, appropriate meal timing could also help to adjust the body clock.

## Data Availability

The datasets generated for this study are available on request to the corresponding author.

## Ethics Statement

The studies involving human participants were reviewed and approved by the Semmelweis University Regional and Institutional Committee of Science and Research Ethics (Ethical approval 170/2016). The patients/participants provided their written informed consent to participate in this study.

## Author Contributions

KE and KK designed the study. ÁS and KE collected the data. ÁS, KE, and RB analyzed the data. KK, ÁS, KE, and RB wrote the manuscript and approved its final version.

## Conflict of Interest Statement

The authors declare that the research was conducted in the absence of any commercial or financial relationships that could be construed as a potential conflict of interest.
