# Supplementary material for: Association of Social Jetlag With Sleep Quality and Autonomic Cardiac Control During Sleep in Young Healthy Men
Source: Front Neurosci. 2019 Sep 6;13:950. doi: 10.3389/fnins.2019.00950 (PMC6742749; doi:10.3389/fnins.2019.00950)
Supplement: Supplementary file 1 [file Data_Sheet_1.docx]

Supplementary Material

**Table S1. Correlation of MSFsc and SJL obtained by the MCTQ and the sleep diary**

|  |  |  |  |  | Correlation | |
| --- | --- | --- | --- | --- | --- | --- |
|  |  | MCTQ | Diary |  | r | p |
| Chronotype (MSFsc) (hh:mm) |  | 04:31 (01:01) | 04:24 (01:01) |  | 0.702 | <0.001 |
| Social jetlag (hh:mm) |  | 01:30 (00:45) | 01:32 (00:53) |  | 0.763 | <0.001 |

Mean (SD), Pearson’s correlation, n=33.

(MCTQ, Munich Chronotype Questionnaire; MSF, midpoint of sleep on free days; MSFsc, MSF corrected for oversleep; SJL, social jetlag)

**Table S2. Time spent awake before sleep on the days of measurements**

|  |  | Lower SJL | |  | Higher SJL | |  |
| --- | --- | --- | --- | --- | --- | --- | --- |
|  |  | workday | free day |  | workday | free day |  |
| time awake (hh:mm) |  | 16:35 (00:15) | 15:21 (00:16) |  | 16:34 (00:09) | 15:07 (00:19) |  |

Time spent awake before sleep on the days (workday and free day) the measurements were performed. Mean (SEM), two-way ANOVA shows neither group (p=0.682) nor group x day effect (p=0.628). (SJL, social jetlag)

**Table S3. 6-sulfatoxymelatonin levels in evening and morning urinary samples**

|  |  |  |  |  |  | Comparisons | |
| --- | --- | --- | --- | --- | --- | --- | --- |
|  | Workday | | Free day | |  | Workday | Free day |
| 6-sulfatoxymelatonin/ creatinine ratio (mmol/nmol) | Lower SJL | Higher SJL | Lower SJL | Higher SJL |  | p | p |
| Evening | 2.143 (0.645) | 2.293 (0.410) | 3.654 (0.715) | 3.988 (1.048) |  | 0.242 | 0.796 |
| Morning | 10.557 (1.354) | 10.791 (1.718) | 16.021 (4.165) | 12.502 (2.318) |  | 0.858 | 0.648 |

Comparison of melatonin levels (urinary 6-sulfatoxymelatonin normalized to creatinine) between groups with lower and higher SJL on workday and free day. Mean (SEM), Mann-Whitney U test, n = 17 and 14 for the group with lower and higher SJL, respectively. (SJL, social jetlag)

**Table S4. Significance levels for the statistical analyses of HRV parameters pNN50 and RMSSD in the course of sleep**

|  |  | Variable | |
| --- | --- | --- | --- |
|  |  | pNN50 | RMSSD |
| Effect |  | p | p |
| group  day  sleep block |  | 0.217  0.207  <0.001 | 0.512  0.219  <0.001 |
| day x group  sleep block x group  day x sleep block  day x sleep block x group |  | 0.285  0.648  0.116  0.015 | 0.380  0.612  0.245  0.059 |

Significance levels for the repeated measures of ANOVA with three levels (group, day, sleep block) used for the analyses of the HRV parameters pNN50 and RMSSD in the course of sleep (see Figure1).
