# Supplementary figures and images for: Association of Social Jetlag With Sleep Quality and Autonomic Cardiac Control During Sleep in Young Healthy Men
Source: Front Neurosci. 2019 Sep 6;13:950. doi: 10.3389/fnins.2019.00950 (PMC6742749; doi:10.3389/fnins.2019.00950)

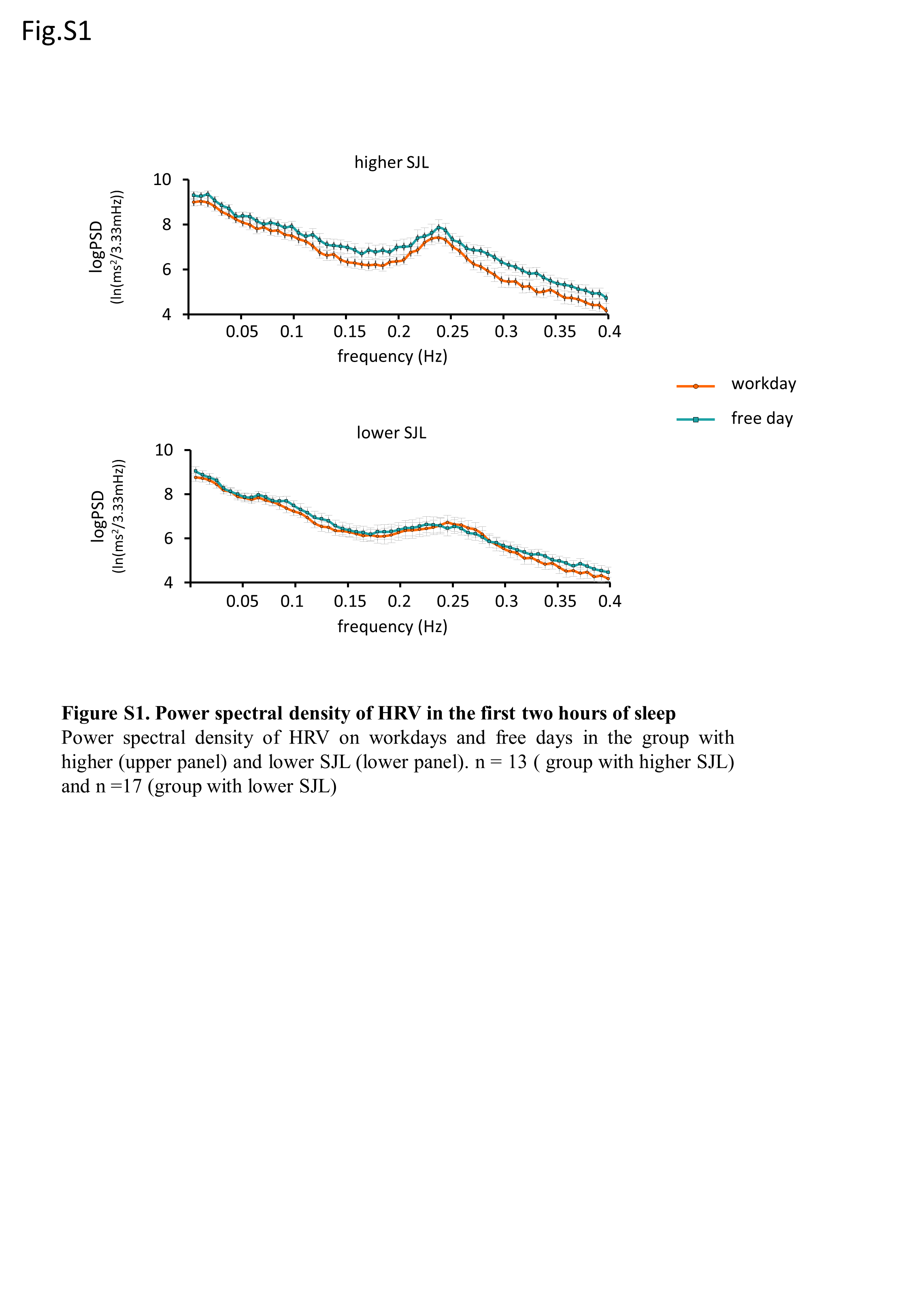

Supplement: Supplementary file 2 [file Image_1.TIF]

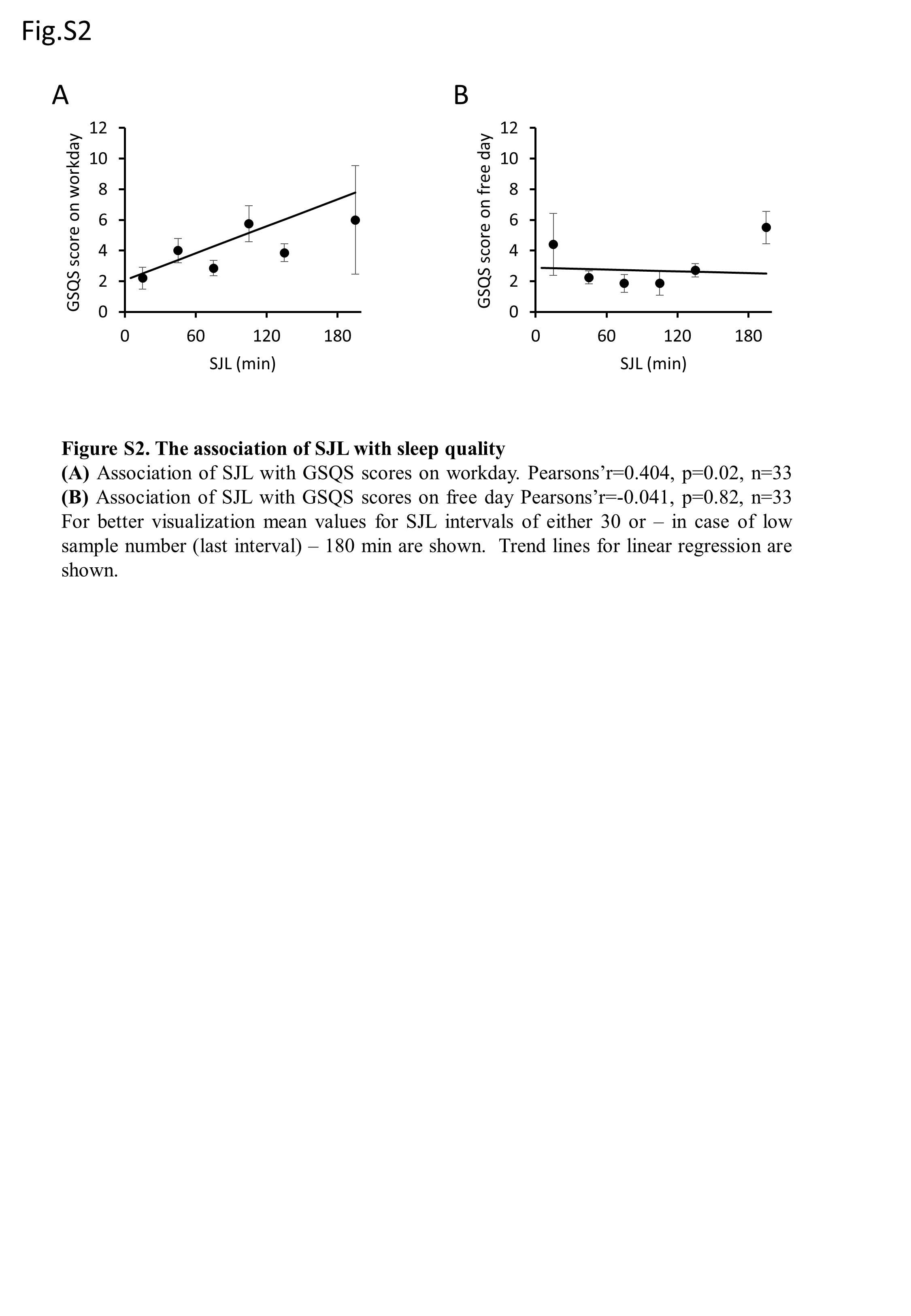

Supplement: Supplementary file 3 [file Image_2.TIF]
